# Towards Efficient Electrically-Driven Deep UVC Lasing: Challenges and Opportunities

**DOI:** 10.3390/nano13010185

**Published:** 2022-12-31

**Authors:** Sergey Nikishin, Ayrton Bernussi, Sergey Karpov

**Affiliations:** 1 Department of Electrical and Computer Engineering and Nano Tech Center, Texas Tech University, Lubbock, TX 79409, USA; 2 Soft-Impact, Ltd., St. Petersburg 194044, Russia

**Keywords:** deep-UV laser diodes, AlGaN, threading dislocations, n- and p-doping, electron leakage, polarization doping, tunnel junction, metalorganic vapor-phase epitaxy, molecular-beam epitaxy

## Abstract

The major issues confronting the performance of deep-UV (DUV) laser diodes (LDs) are reviewed along with the different approaches aimed at performance improvement. The impact of threading dislocations on the laser threshold current, limitations on heavy n- and p-doping in Al-rich AlGaN alloys, unavoidable electron leakage into the p-layers of (0001) LD structures, implementation of tunnel junctions, and non-uniform hole injection into multiple quantum wells in the active region are discussed. Special attention is paid to the current status of n- and p-type doping and threading dislocation density reduction, both being the factors largely determining the performance of DUV-LDs. It is shown that most of the above problems originate from intrinsic properties of the wide-bandgap AlGaN semiconductors, which emphasizes their fundamental role in the limitation of deep-UV LD performance. Among various remedies, novel promising technological and design approaches, such as high-temperature face-to-face annealing and distributed polarization doping, are discussed. Whenever possible, we provided a comparison between the growth capabilities of MOVPE and MBE techniques to fabricate DUV-LD structures.

## 1. Introduction

Light sources emitting in the deep ultraviolet (DUV) wavelength range are needed in many areas including the germicidal decontamination of air and water, detection and qualitative determination of toxic and non-toxic gas concentrations, non-line-of-sight communication, charge management of gravitational effects, and skin-safe disinfection. For example, UVC light below 280 nm wavelength has a strong biocidal effect against protozoa, bacteria, viruses, and molds, destroying or inactivating microorganisms so that they can no longer multiply. Light at wavelengths near 265 nm is the most effective for producing this effect [1,2]. Light at 280 nm wavelength and below is also needed for non-line-of-sight urban communication applications [3] and can be potentially used in deep UV lithography. UVC light is crucial for applications including the safeguarding of mobile and stationary water supplies, mobile water purification facilities, general municipal water treatment, the treatment of public and private swimming facilities, air treatment of enclosed facilities, and biomedical sanitation [2,4]. It has been recently shown that skin disinfection by UV radiation with wavelengths shorter than 240 nm is much more attractive than radiation in the range of 250–280 nm, since it is mainly absorbed in the upper non-living layer of the skin [5].

III-nitride compounds and their alloys, particularly direct bandgap AlN, GaN, and AlGaInN, are very promising semiconductors for realization of such DUV sources, light-emitting diodes (LEDs) and semiconductor lasers with emission spanning from 210 to 330 nm wavelengths. These LEDs and lasers will replace fragile and unreliable mercury-based bulbs and tubes with rugged, long-life, semiconductor-based devices [6]. Lasers are more preferable due to a higher irradiance, lower light beam divergence, and superior focusing capability of the emitted light, which is especially important in medical applications. Note that lasers can be operated under optical, electrical (electrically-driven), or electron beam (electron gun) excitation. All these three approaches have been already utilized to demonstrate III-nitride-based DUV lasers [7,8,9,10,11,12,13,14,15,16,17,18,19,20,21,22,23]. Undoubtedly, electrically-driven laser diodes (LDs) that can deliver high optical output powers at specified wavelengths and, at the same time, are robust, reliable, and portable, are more desirable due to their durability and compactness when compared to optically- or electron-beam-pumped counterparts. Therefore, the electrically-driven LDs will be the main focus of this review.

**Figure 1:**
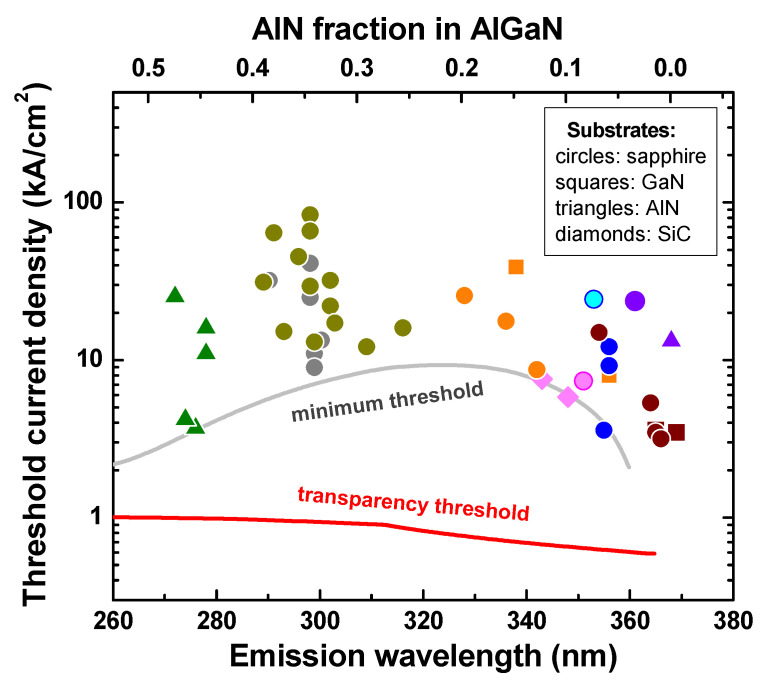
Experimental threshold current densities of UV laser diodes fabricated on various substrates (symbols) vs. oscillation wavelength [24,25,26]. The grey line is an approximation for the minimum threshold current density drawn as a guide to the eye. The red line is the calculated transparency threshold (see text for more detail). The top scale displays the compositions of bulk AlGaN corresponding to the wavelengths indicated in the bottom scale.

Figure 1 summarizes reported experimental threshold current densities (symbols) of UV laser diodes for different emission wavelengths. The data were obtained from [24,25,26]. Detailed description of the results by various research groups is given in [24]. The approximation for the minimum threshold current density is shown in Figure 1 as a guide to the eye by the grey curve. The red solid line in Figure 1 is the simulated transparency threshold, i.e., the current density corresponding to the onset of optical amplification of the laser diode. For simplicity, the transparency threshold current was calculated from the equation *F_n_* – *F_p_* = *E_g_*, where *E_g_* is the energy gap of bulk AlGaN alloy and *F_n_* and *F_p_* are the quasi-Fermi levels of electrons and holes, respectively. In the calculations, the electron and hole densities were assumed to be identical. Shockley–Read–Hall recombination coefficient *A* = 10^8^ s^−1^, Auger recombination coefficient *C* = 2.3 × 10^−30^ cm^6^/s [27], and radiative recombination coefficient *B* varied from 1.6 × 10^−11^ to 2.2 × 10^−11^ cm^3^/s, depending on the AlGaN composition, were used for the transparency threshold current estimations.

**Figure 2:**
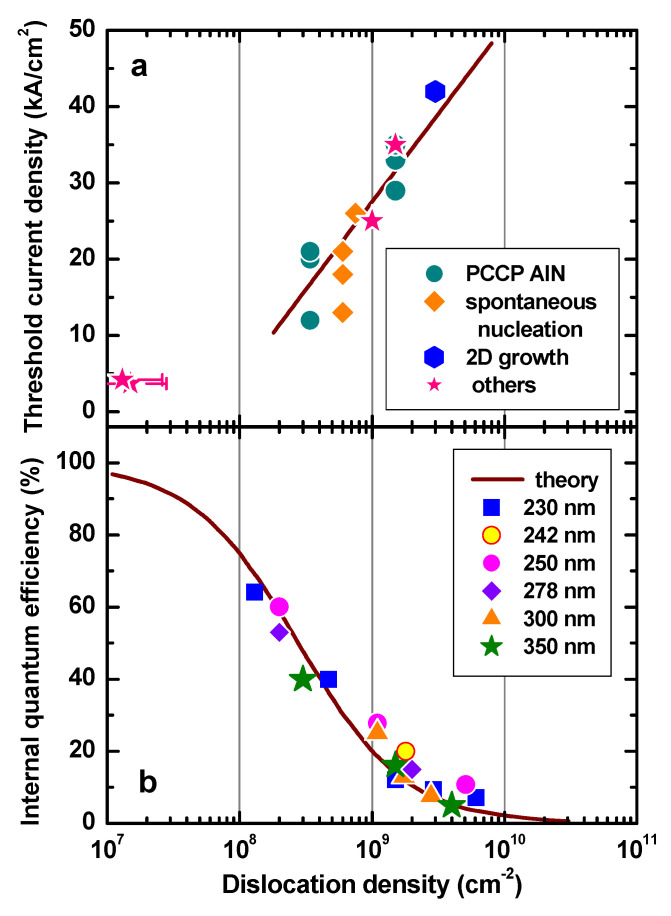
(**a**) Experimental threshold current densities of UV laser diodes (symbols) fabricated by various approaches, including growth on concave-convex patterned AlN (PCCP AlN) vs. TDD [25,28,29,30]; the straight line approximates the data. (**b**) Measured internal quantum efficiency (symbols) of UV-LEDs emitting at various wavelengths as a function of TDD in the LED structure [31,32,33]; the solid line is the theoretical curve calculated by the model described in [34].

The following important conclusions can be deduced from Figure 1. First, there is a tendency of lower threshold current density towards shorter wavelengths in the UVB (280–315 nm) and UVC (200–280 nm) spectral ranges. This is apparently due to the fact that practically all the laser diodes in the UVC range were fabricated on bulk or quasi-bulk AlN templates/substrates. This produces extra strain in the LD structures, which increases with the wavelength. Second, there is a large gap between the transparency threshold and the minimum threshold current density, sometimes approaching an order of magnitude difference. This gap has a distinct correlation with threading dislocation densities typical for AlGaN alloys, which were shown in Figure 1 of the review reported in [24]. This correlation reveals a very critical effect of threading dislocation density (TDD) on the threshold current density of DUV-LDs, which was further confirmed by direct measurements reported in [25,28,29,30], see Figure 2a (TDD for the lowest threshold current density shown in this figure was not reported in [25], but we estimated TDD in the range of ~10^6^–10^7^ cm^−2^). The efforts aimed at TDD reduction, thus improving the threshold current density, will be discussed in more detail in Section 2.

The goal of the present study is to review the main challenges faced in the fabrication of electrically-driven AlGaN-based UVC LDs using different technologies, the possible solutions to overcome (or at least partially remediate) these issues, and to discuss remaining unresolved problems. Section 2 of this review considers a crucial role of threading dislocations in determining internal quantum efficiency (IQE) of light emission and its correlation with the threshold current density of LDs. The problems of doping of Al-rich AlGaN alloys in view of the LD electrical design are discussed in Section 3. In Section 4, we discuss specific features of UVC LD design, including the problem of carrier losses by electron leakage into the p-side of the device structure and the improvement of uniformity of hole injection in the active region. A summary of this study and an outlook of future research perspectives are provided in Section 5. 

## 2. Material Quality and Its Impact on Internal Quantum Efficiency and Threshold Current Density of UVC Laser Diodes

Threshold current density of any LD is primarily controlled by corresponding non-equilibrium carrier density in the active region of the device. In turn, the carrier density is determined by recombination dynamics and competition between radiative and non-radiative recombination channels. Therefore, internal quantum efficiency (IQE) of light emission and threshold current density of an LD closely correlate to each other. 

Figure 2b shows the dependence of IQEs of various UV LEDs on threading dislocation density (TDD) in the wafers, primarily of edge- and mixed-types. Despite different emission wavelengths and different LED structure designs, all the data form likely a universal trend, which is well approximated by a theoretical curve. This means that TDD is the dominant factor determining the IQE, and that other recombination channels, i.e., Shockley–Read–Hall (SRH) and Auger recombination, are of secondary importance. Therefore, TDD reduction is vital for both IQE and threshold current density in the UV laser diodes (see Figure 2).

The lowest TDD shown in Figure 2b is about 1–2 × 10^8^ cm^−2^. At such a TDD, blue InGaN-based LEDs provide IQE exceeding 80% [35], whereas only 60% is attained in UV LEDs in practice (see Figure 2b). To reach IQE of 80%, TDD should be reduced to 5–6 × 10^7^ cm^−2^. This difference originates from a weaker impact of carrier localization by composition fluctuations in AlGaN alloys compared to InGaN ones. 

Already, earlier studies revealed a crucial role of AlN buffer layers commonly employed for growing light-emitting structures on sapphire substrate by metalorganic vapor-phase epitaxy (MOVPE). Considerable reduction of TDD from 5 × 10^9^ to 2 × 10^8^ cm^−2^ was demonstrated by using the migration-enhanced MOVPE (MEMOCVD^®^) technique [33,36]. Pulsed-flow temperature/pressure-modulation MOVPE was used in the fabrication of 4.8 μm multi-layer AlN buffer layer with the edge dislocation density of 1.8 × 10^9^ cm^−2^ [37]. Optimization of the growth conditions in this technique allowed further TDD reduction down to 5 × 10^8^ cm^−2^ [38]. 

Remarkable progress was attained by increasing MOVPE growth temperature up to 1400 °C and using a modulated V/III ratio, which enabled fabrication of thick, 9 μm, AlN buffer layers with the edge-type dislocation density of 3 × 10^8^ cm^−2^ [39]. However, a real breakthrough was achieved by a combination of the high-temperature growth with patterning of sapphire substrate by groove etching followed by epitaxial lateral overgrowth (ELO) [40,41]. As a result, voids in the underlying AlN layer were formed at the initial stages of the growth. Nevertheless, a proper choice of the direction of grooves etching in the sapphire substrate produced high-quality overgrowth of AlN with the mean TDD of 4 × 10^7^ cm^−2^ [40]. Such a TDD was potentially capable of providing IQE ~80% in the light-emitting structure. However, a disadvantage of the approach was the large thickness of the whole AlN layer, ~15 μm in total, which makes the epitaxial growth too expensive.

Molecular beam epitaxy (MBE) is the alternative method for growing DUV laser structures based on AlGaN semiconductors. Several approaches have been applied to reduce the dislocation density in AlN and AlGaN layers grown on different substrates using plasma-assisted (PAMBE) and gas source (GSMBE) with ammonia [22,23]. One of the MBE advantages is in situ monitoring of the growth mode, two-(2D) or three-dimensional (3D), using the reflection high energy electron diffraction (RHEED) technique. Because of a large lattice mismatch with AlGaN, the commonly used substrate, (0001) sapphire, must be overgrown with an AlN buffer layer for subsequent growth of the device structure. At the initial stage of the growth, the dislocation density of AlN/sapphire exceeds 10^11^ cm^−2^ [12]. It was shown that the TD dislocation density could be controlled and reduced in the AlN buffer layer by using a migration-enhanced PAMBE [42]. Combination of high-temperature growth of AlN layers, nucleated by a migration-enhanced PAMBE on (0001) sapphire substrate, with insertion of 3−6 nm thick 2D/3D-grown GaN layers and additional deposition of AlGaN/AlN superlattices on top, enabled the reduction of screw and other TD densities in the buffer layer to 4–7 × 10^8^–2 × 10^9^ cm^−2^ [12,43]. It was shown that using GSMBE with ammonia resulted in densities of screw dislocations as low as 3 × 10^8^ cm^−2^ in AlN/AlGaN short-period superlattices (SPSLs) grown on Al-polar (0001) AlN substrates, which is an order of magnitude lower than TDD obtained by the growth of the same structures on AlN/c-Al_2_O_3_ buffer layers [44]. At such TDD, the maximum achievable IQE is less than 20%, and the threshold current density of an LD should exceed ~35 A/cm^2^ (see Figure 2a,b).

Recently, a new approach to the fabrication of low-dislocation AlN buffer layers was developed, based on high-temperature face-to-face annealing (HT-FFA) [45,46]. The starting point of the technique is the deposition of poly-crystalline AlN on c-plane sapphire substrates by radio-frequency sputtering of either sintered AlN or high-purity Al targets in the N_2_ ambience (the choice of the target controls the dominant polarity of the sputtered film [46]). Then, two wafers prepared by sputtering were placed face-to-face together and annealed at temperatures of 1600–1750 °C for up to 48 h in N_2_ environment. In the course of the annealing, the interface between the two wafers formed a flat polarity-inversion boundary, while the microstructure of the AlN films exhibited a solid-phase transformation from a columnar one to a single-crystal domain with residual micro-voids [47]. After separating one of the sapphire substrates, the template becomes ready to be used for epitaxial growth of the device structure.

The HT-FFA technique provided AlN templates with TDD as low as 2 × 10^8^ cm^−2^. Further TDD reduction to 1.5 × 10^8^ cm^−2^ was accomplished by using thermo-cycling during the annealing stage [46]. The use of double HT-FFA with a second AlN film deposition on the already annealed template followed by a secondary annealing resulted in the reduction of TDD down to 4.3 × 10^7^ cm^−2^ [46].

To summarize, the HT-FFA technique looks quite advantageous for fabrication of low-dislocation AlN templates for deep-UV light emitters. However, there are still some challenges hindering its wide application. First, the surface of the templates is yet insufficiently smooth for subsequent epitaxial growth. Second, there is a huge oxygen incorporation into the AlN layer during the template preparation, which leads to (i) inducing columnar MOVPE growth of device structure and (ii) decreasing the lattice constant of the AlN because of the smaller atomic radius of oxygen when compared to nitrogen [48]. The latter factor should be accounted for in strain management of the device structure. In view of the above challenges, alternative approaches combining HT-FFA with epitaxial lateral overgrowth (ELO) on patterned sapphire substrates are now intensively studied [48].

The above discussion suggests that a direct way for substantial TDD reduction seems to be the use of AlN substrates made of bulk AlN and characterized by a low TDD of ~10^3^–10^4^ cm^−2^. Compressively strained AlGaN layers can actually be grown on bulk AlN without stress relaxation accompanied by new dislocation generation far above the Matthews–Blakeslee critical thickness [49,50]. However, the absence of stress relaxation results in a very strong bowing of the whole wafers, limiting their further use for device processing. To date, an optimal strategy of strain management during the growth of LED and LD structures on bulk AlN substrates has not yet been developed [50]. In order to utilize the effective method of stress relaxation by Si-doping induced dislocation inclination [51], it is necessary to maintain a certain TDD in the initial AlN buffer layer. This optimal TDD, capable of producing stress relaxation to avoid the wafer bow, will eventually determine the maximum achievable IQE of the light-emitting device and affect, subsequently, the achievable threshold current densities of the UVC LD.

## 3. Low-Resistive AlGaN Layers and Doping Problems

### 3.1. N-Type Doping of AlGaN

N-type AlGaN plays two main roles in the LD structures. First, it supplies electrons to the active region from a heavily-doped n^+^-AlGaN cladding layer. Second, the same cladding layer provides appropriate lateral current spreading, which is necessary in view of typical on-one-side contact electrode geometry. Particularly, the second role is required to maximize the electrical conductivity of the n^+^-AlGaN claddings. 

**Figure 3:**
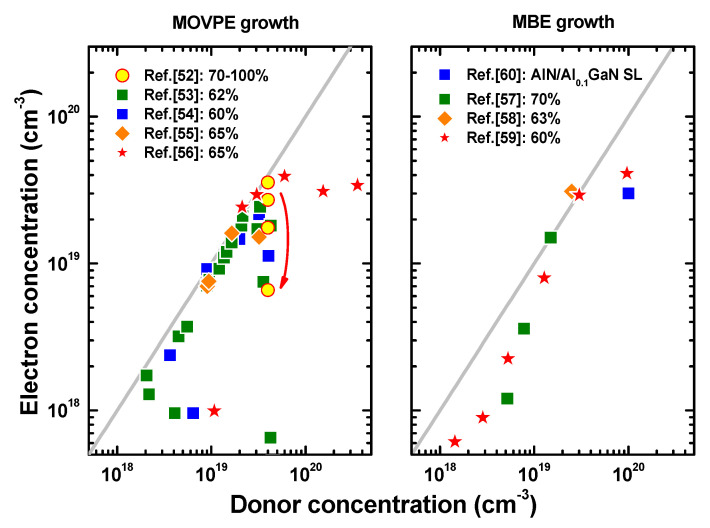
Electron concentration vs. Si-donor concentration in n^+^-AlGaN alloys grown by MOVPE (**left**) and MBE (**right**) techniques. Symbols are data from various sources (see text). Grey solid lines correspond to equal electron and donor concentrations. The arrow in the left plot indicates the decrease of the electron concentration with the AlN molar fraction reported by Nakarmi et al. [52]. Inset legends on both plots indicate the percentage of Al in the AlGaN alloys [53,54,55,56,57,58,59,60].

Figure 3 shows Hall’s electron concentrations in the n^+^-AlGaN alloys as a function of incorporated Si-donor concentrations using MOVPE and MBE growth techniques [52,53,54,55,56,57,58,59,60]. One can see that the maximum electron concentration achieved by both MOVPE and MBE to date is ~4 × 19^19^ cm^−3^ [56,59]. Most of the reported MOVPE data show a dramatic drop of the electron density starting from [Si] = ~4−5 × 19^19^ cm^−3^, attributed to the massive generation of point defects, of which the origin is still under debate [55]. Optimization of the growth temperature and the V/III ratio aimed at reduction of the point defect density enables avoiding of the drop [55,56]. However, further increase in the electron density with Si-donor concentration has not been achieved yet. Some data points in the range of 10^18^ < [Si] < 10^19^ cm^−3^ exhibit considerable deviations from the grey line in Figure 3. This fact may be attributed to a reduction of the electrical conductivity by parasitic impurities, mainly by carbon, being a compensating acceptor in MOVPE [61], and mainly by oxygen in the case of MBE [62].

Recently, the advantages of using Ge instead of Si have been demonstrated for n-type doping of Al_0.3_Ga_0.7_N [61]. Ge donors were capable of producing three-times higher maximum electron concentration than Si. However, the positive effect of n-type doping with Ge has not yet been demonstrated in the case of Al-rich AlGaN alloys.

A special approach to n-type heavy doping is the use of digital alloys instead of bulk Al-rich AlGaN. It was shown, in particular, that using 1.2 nm AlN/0.5 nm Al_0.1_Ga_0.9_N short-period superlattice (SPSL), equivalent to *x* = 0.72 in a uniform Al*_x_*Ga_1−*x*_N alloy, doped with Si up to 1 × 10^20^ cm^−3^ enabled achievement of a mean electron concentration of 3 × 10^19^ cm^−3^ and an in-plane mobility of 10–20 cm^2^/V·s in the SPSL [62]. It should be noted, however, that the mobility normal to the epitaxial layers was about an order of magnitude lower. Nevertheless, the above approach is quite suitable for maintaining excellent lateral current spreading in an LD structure by including such n-doped digital alloys.

**Figure 4:**
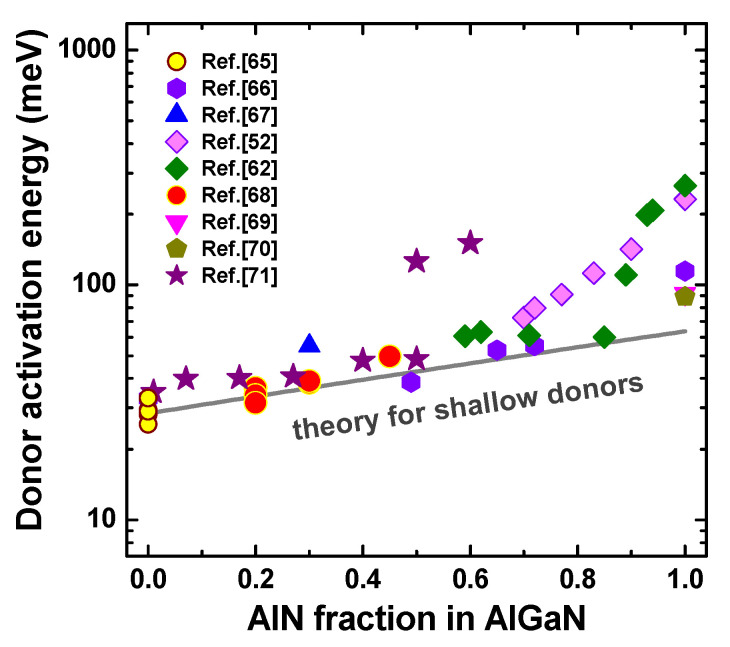
Experimental low-concentration activation energies of the Si donor vs. AlGaN composition corrected for particular donor concentrations (symbols). The grey line is the theoretical curve calculated for shallow-donor states [52,62,65,66,67,68,69,70,71].

Except for self-compensation, formation of DX-states instead of shallow-donor ones may increase substantially AlGaN resistivity because of a dramatic increase in the donor activation energy. For Si, the most frequently-used donor impurity in Al*_x_*Ga_1−*x*_N, ab initio calculations predict the onset of DX-state formation at the AlN molar fractions *x* varying from 0.22 [63] to 0.94 [64]. In order to distinguish between the DX- and shallow-donor states of Si in AlGaN, data on the donor activation energy from various sources (see refs. [65,66,67,68,69,70,71]) were analyzed in [72]. Direct plotting of the activation energies as a function of the AlGaN composition did not reveal any distinct trend because of the large scattering of the experimental data obtained for very different donor concentrations in various samples. After a correction was made to evaluate the low-concentration (single donor) activation energies, all the experimental points, except for two outliers, remained slightly above or very close to the line representing the theoretical activation energy of the shallow donors (see Figure 4). The theoretical curve was obtained by using the variational approach, accounting for anisotropy of both electron effective mass and static dielectric constant in the AlGaN alloys [72].

An important conclusion obtained from Figure 4 is that the shallow-donor states of Si in AlGaN are observed practically over the entire range of AlGaN compositions, including pure AlN. As the DX-center and shallow-donor are alternative electronic states of the same impurity, i.e., they cannot coexist in the bulk of the semiconductor, we can conclude that it is unlikely that DX-states are formed in AlGaN doped with Si at any alloy composition. A gradual deviation of the measured activation energies from the shallow-donor line at *x* exceeding 0.7–0.8 may be attributed to a substantial increase of the residual oxygen and carbon concentrations in the alloys, which might become comparable to the concentration of Si.

### 3.2. P-type doping of AlGaN 

**Figure 5:**
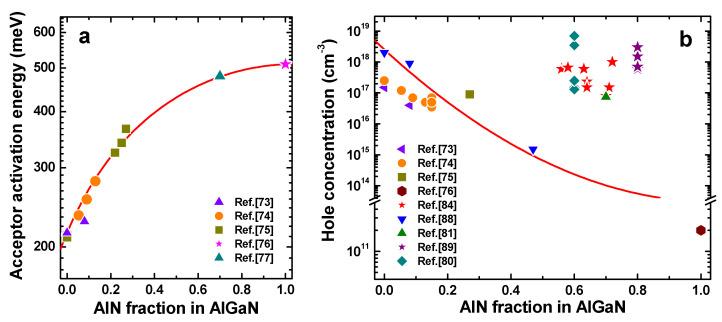
Experimental low-concentration activation energies of Mg acceptors (**a**) and hole concentrations in AlGaN alloys and AlGaN/AlN superlattices (**b**) vs. bulk AlGaN composition (symbols). Solid lines correspond to a polynomial fitting approximation of the experimental activation energies in (**a**), and to an exponential decay demonstrating the trend in hole concentration variation, accounting for the composition-dependent activation energy, in (**b**). The asterisk symbols correspond to the data obtained using AlGaN/AlN superlattices [73,74,75,76,77,80,81,84,88,89].

Magnesium is the only practical impurity used as a dopant in III-nitride semiconductors, providing relatively “shallow” acceptor levels. Figure 5a summarizes the experimental low-concentration acceptor ionization energies as a function of AlGaN composition [73,74,75,76,77]. To obtain the low-concentration energies, the actual ones reported in the cited papers were corrected for each acceptor concentration. The solid line shown in Figure 5a represents the data approximation by a quadratic polynomial equation: *E_A_*(*x*) = 510*x* + 215(1 − *x*) + 270*x*(1 − *x*) [meV]. 

A considerable increase in the ionization energy of Mg acceptors in Al-rich AlGaN observed in Figure 5a results in a low hole concentration in such alloys. In most reports [73,74,75,76], the room-temperature (RT) hole concentration does not exceed ~1–2 × 10^18^ cm^−3^ (see Figure 5b and [78]). At a huge Mg concentration incorporated into the alloy, ranged between 10^19^ and 10^20^ cm^−3^, the RT hole concentration may, however, approach the value of ~1 × 10^19^ cm^−3^ (see Figure 5b, Table 3 in [79], and [80], respectively). Such an increase in the hole concentration is accompanied by a reduction of the acceptor ionization energy down to ~30–50 meV and a dramatic reduction in the hole mobility [81,82], attributed to impurity-band conduction. As a result, the overall electric conductivity varies weakly with the Mg concentration. Therefore, the growth of low-resistive bulk p-type layers in UVC LDs, necessary to lower their operating voltages, is still a quite problematic and challenging task.

Application of Mg-doped AlN/(AlGaN SPSLs) grown by MBE [60,83,84,85,86,87,88] and MOVPE [89,90,91,92] is a solution which enables (i) obtaining comparably high concentrations of holes averaged over the superlattice period (see Figure 5b, where asterisks correspond to the data obtained by SPSLs) and (ii) significantly reducing the acceptor activation energy. For example, in MBE-grown AlN/Al*_x_*Ga_1−*x*_N (0.03 < *x* < 0.08) SPSLs with 0.5–0.8 nm wells and 1.5–2 nm periods, the average AlN concentration could be changed in the range of *y*_av_ = 0.5–0.8 [81,88] by variation of the well-to-period width ratio. For MOVPE-grown AlN/Al_0.75_Ga_0.25_N SLs with 4 nm wells and 5.3 nm period, *y*_av_ = 0.8 can be achieved [89]. The average Mg concentration in these SPSLs did not exceed 3.5 × 10^19^ cm^−3^, which did not affect the material quality. The mean hole concentration in SPSLs might be as high as 1–3 × 10^18^ cm^−3^ at a rather high mean Al content, as is seen from Figure 5b. The temperature dependence of the hole concentration corresponded to the effective activation energy of ~40–67 meV, i.e., much lower than that of Mg-doped Al_0.8_Ga_0.2_N alloys (see Figure 5a). Similar temperature dependencies with low effective activation energies were also reported for bulk AlGaN:Mg and GaN:Mg layers by various research groups [92,93,94,95]. However, they were attributed to extremely high doping level in the semiconductors. Weak variation of the hole concentration with temperature in SPSLs makes them very attractive for fabrication of thermally stable electric contacts with low specific contact resistance [88]. However, the p-type SPSLs have the same disadvantage as the n-type SPSLs, i.e., a lower vertical electrical conductivity, as compared to the lateral one [96].

Mg–O co-doping is an interesting approach to obtain p-type conduction in GaN and AlN [97,98,99]. In particular, an ab initio study of Mg–O complexes in AlN [99] predicted the acceptor activation energy to be at least 230 meV lower than that of Mg itself in AlN. Experimentally, this approach was validated using GaN and Al_0.08_Ga_0.92_N alloys grown by ammonia MBE [100] and MOVPE [101]. It was shown that the acceptor activation energy could be reduced to 145–135 meV in the GaN co-doped with Mg–O and the room-temperature hole concentration of ~2–3 × 10^18^ cm^−3^ was reached at [Mg] ~ 1–2 × 10^20^ cm^−3^. A similar hole concentration was measured in Al_0.08_Ga_0.92_N alloys at the same density of Mg impurities [100]. Note that the hole concentration in ammonia MBE-grown GaN and Al_0.08_Ga_0.92_N depends on both NH_3_ flux and Mg/O ratio, which makes controlling the growth process more difficult. Nevertheless, it would be quite tempting to apply p-type co-doping to AlGaN alloys with high Al contents.

Considering the above issues related to appropriate p-doping of the LD structures, the most effective way to attain high hole concentrations is based now on distributed polarization doping (DPD) in graded-composition AlGaN alloys. Its application to forming layers with sufficiently high hole densities was suggested theoretically in [102] and demonstrated experimentally in [103]. In UVC LD structures, DPD is almost exclusively employed in the electron-blocking layers (EBLs) made of an AlGaN alloy with Al content decreasing along the hexagonal axis of the crystal [18,28,104,105,106]. Generally, the hole concentration in a graded-composition Al*_x_*Ga_1−*x*_N layer coherently grown on an Al_0.6_Ga_0.4_N base can be estimated as follows: *p* = *C*(Δ*x*/*d*), where Δ*x* is the total composition variation across the layer, *d* is its thickness, and *C =* 6.9 × 10^20^ nm/cm^3^ is the coefficient accounting for contributions of both spontaneous and piezo-polarization to hole generation. For typical LD structures [28,104,105,106], the composition gradient Δ*x*/*d* is 1–2 × 10^−3^ nm^−1^, providing the estimated hole concentration of 0.65–1.20 × 10^18^ cm^−3^ only. To make the concentration greater than 10^19^ cm^−3^, the DPD layer thickness should be reduced to ~30 nm. Such a reduction may, however, lead to cracking of the DPD layer during growth, limiting the DPD capability. This issue and other limiting factors require further detailed investigations.

An alternative approach capable to overcome the problem of insufficient p-doping in UVC LD structures is the use of tunnel junction (TJ) followed by the deposition of an n-type contact layer, the Ohmic contact to which can be much more easily formed when compared to p-AlGaN materials. This approach is discussed below in Section 4.3.

## 4. Laser Structure Design 

This section is focused on specific problems related to structure design and optimization for UVC LDs. Special attention is paid to electron leakage into the p-side of an LD structure, a challenge which has not yet found an acceptable solution. Other important factors are discussed as well, including the choice of the active region design, improvement of lateral current spreading in the p-type layers, and application of TJs to overcome the problem of insufficiently-high p-doping level.

### 4.1. Typical UVC Laser Diode Structures 

**Figure 6:**
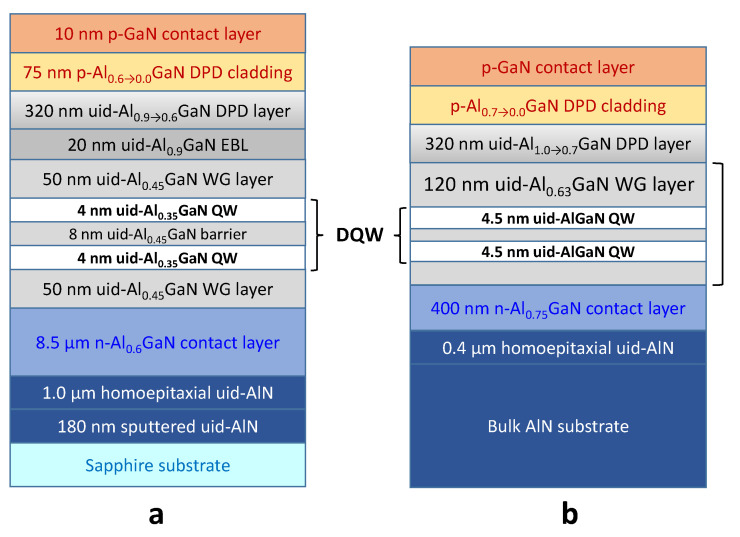
Schematic illustrations of typical structures of UVC LDs grown on a sapphire [24] (**a**) or a bulk AlN [25,26] (**b**) substrate. Unintentionally-doped epitaxial layers are marked by the acronym ‘uid’.

Currently, there are only a limited number of reports demonstrating operation of electrically-driven UVC LDs. Detailed analysis of those reports suggests two typical LD structures: one intended for growth on a sapphire substrate and another suitable for growth on a bulk AlN substrate (see Figure 6). 

The principal difference between the structures is the thickness of the n-AlGaN contact layers. When growing on sapphire, much thicker n-AlGaN contact layers are needed to reduce TTD in the active region by dislocation inclination in Si-doped AlGaN followed by their annihilation. In the structures shown in Figure 6, e.g., the thickness of the layers differ almost by a factor of 20. However, the thickness of the n-AlGaN contact layer should also be optimized for growth on an AlN substrate in order to provide a good lateral spreading of the electric current in the ridge-type LD, which is commonly used in the fabrication of the devices on both types of substrates. 

For both structures shown in Figure 6, a rather narrow, 100–120 nm, waveguide (WG) layer contains sufficiently wide AlGaN double-quantum wells (DQWs) aimed at increasing the optical confinement factor. In the earlier demonstrations of injection-driven UVC LD [104,105], a 9 nm AlGaN SQW was used instead of the DQW. Because of asymmetric distribution of the AlGaN composition and cladding thicknesses in the LD structure, the DQWs are frequently shifted from the center of the WG towards n-AlGaN contact layer in order to increase the optical confinement factor (see Section 4.3) [25,26]. 

In both LD structures shown in Figure 6, graded-composition DPD layers serve simultaneously as an EBL and a hole-injector layer. This two-purpose role resulted in the necessity of using two different graded-composition materials. One, adjacent to the active region, had a higher Al content and was unintentionally doped. Another, placed next to the p-GaN contact layer, had a higher composition gradient and was additionally doped with Mg.

It should be noted that a minimum threshold current density of 3.7–4.3 kA/cm^2^ [25,26] and room-temperature continuous-wave operation of lasers [26] have been achieved by using the structures grown on AlN substrates. This result agrees well with our consideration of the role of TDD for the threshold current control discussed in Section 2. 

### 4.2. Electron Leakage into P-Layers of Device Structures 

Because of large band offsets, low values and large differences between electron and hole mobilities, and extremely high operating current density, the UVC LDs are expected to suffer substantially from electron leakage into the p-side of the device structure. In order to prevent the lasers from this leakage, an EBL layer is conventionally inserted into the structure between the active region and the p-side waveguide layer. In deep-UV LEDs and LDs, the EBL is conventionally made of AlN capable of providing the largest potential barrier for electrons outgoing from the device active region. However, when the Al content in the active region is high enough, the potential barrier formed by the EBL becomes insufficiently high, thus enhancing the electron leakage. Since there are no EBL materials capable of producing a larger potential barrier than that formed by AlN, the leakage enhancement at shorter emission wavelengths plays a fundamental role.

In order to study the electron leakage quantitatively, we have chosen a simple structure with an unintentionally-doped single 1.5 nm-thick Al*_x_*Ga_1−*x*_N quantum well (SQW), 7 nm-thick Al*_y_*Ga_1−*y*_N last barrier (*y* = *x* + 0.15), and 10 nm-thick AlN EBL, all sandwiched between thick n- and 200 nm p-type cladding layers doped with Si and Mg up to the concentrations of 1 × 10^19^ and 5 × 10^19^ cm^−3^, respectively. The cladding layers had the AlN molar fraction of 0.8 at the SQW compositions *x* ≤ 0.65 and it was equal to that of the last barrier at *x* > 0.65. The simulated structure, which did not resemble an LD one, was chosen intentionally in order to avoid some complications in its operation caused by electric potential redistribution between the active region, waveguide layers, and claddings. The operational behavior of this structure was believed to best reproduce all the impacts of intrinsic AlGaN properties on the electron leakage. 

**Figure 7:**
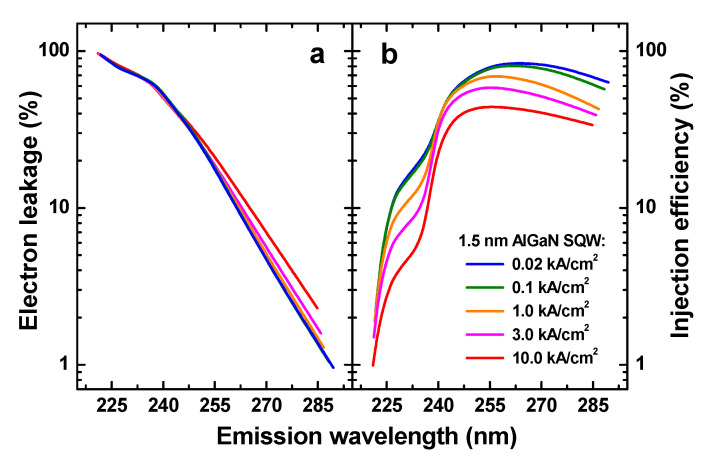
Percentage of electron leakage current of the total current flowing through the AlGaN SQW structure (**a**) and injection efficiency in the SQW (**b**) as a function of emission wavelength.

Figure 7 shows the simulated fraction of the electron-leakage current of the total current flowing through the above SQW structure and the efficiency of carrier injection into the SQW vs. emission wavelength. The curves were obtained by varying the Al*_x_*Ga_1−*x*_N composition in the SQW at different operating current densities ranging from 20 A/cm^2^ (typical for LED operation) to 10 kA/cm^2^, nearly corresponding to the minimum threshold current densities attained in DUV-LDs. One can see that the leakage depends exponentially on the wavelength, varying rather weakly with the current density. The leakage can be reduced below 10% only at wavelengths longer than ~260 nm. At wavelengths shorter than ~240 nm, the electron leakage is predicted to exceed 60% of the total current. 

In contrast to the leakage current, the injection efficiency (IE) saturates at wavelengths longer than ~250–255 nm, whereas the saturation level depends on the current density. In particular, IE does not exceed 45% at the current density of 10 kA/cm^2^, but it may be as high as 80% at 100 A/cm^2^. At wavelengths shorter than ~240 nm, IE decreases dramatically, especially at high current densities. The knees in the curves shown in Figure 7, which are observed at ~240 nm wavelength, correlate with the changes in the SQW structure design: to achieve short wavelengths, the Al content in the cladding layers was increased together with that of the last barrier. 

Considerable electron leakage is a very serious problem in UVC LDs, affecting both threshold currents and slope efficiencies of the oscillating devices. As discussed above, electron leakage is fundamentally related to the intrinsic material properties of AlGaN alloys and structures. Until EBL materials more suitable than AlN are found, remedies aimed at electron leakage suppression to be usually considered are (i) the use of multi-quantum-barrier EBLs [107] and (ii) the application of DPD to increase the potential barrier formed by the EBL [102]. In particular, the latter approach is already used in practice: actually, the DPD graded-composition AlGaN layers in the LD structures reported in [18,28,104,105,106] serve simultaneously as EBLs and hole injectors. 

Another approach to electron leakage suppression, rarely discussed in the literature, is the use of N-polar LD structures. Due to specific distribution of polarization charges at the structure interfaces, a natural high potential barrier to electrons is formed by conventional EBL layers [108]. This approach is not seriously regarded as a growth technology of N-polar structures still requires substantial improvement, primarily to avoid material contaminations with parasitic impurities [109,110].

### 4.3. Active Region Design and P-Type Ohmic Contacts

Typical active regions of DUV-LEDs incorporate multi-quantum wells (MQW) to obtain high IQE of light emission and improved device reliability. This approach is not suitable for LDs because of the non-uniform distribution of hole concentrations among the QWs. Indeed, in InGaN-based MQW structures, holes are primarily injected into upper QW adjacent to the EBL and p-layers [111]. In the case of LD structure, such non-uniformity of injection would produce optical gain in the upper QW only, whereas other wells would absorb photons. Since the non-uniformity in the hole injection is enhanced in the AlGaN-based structures as compared to InGaN-based ones, UVC LDs typically contain either SQW [104,105] or DQW [18,28,106,112] active regions (see Section 4.1). This provides an optical confinement factor of 2.9% for 9 nm SQW active region [104] and 3.5% for 4 nm DQW ones [18], which is insufficiently high to further reduce the threshold current density of UVC LDs. 

An additional limitation to the optical confinement factor originates from the use of p-type DPD EBL and cladding layers. Because of their small thicknesses when compared to the n-type waveguide and cladding layers, a highly asymmetric waveguide structure is typically formed in UVC LDs. As a result, the position of the SQW or DQW active region inside the structure must be carefully optimized in order to increase the optical confinement factor. Such optimization has provided, in particular, an improvement up to 6% by using 4.5 nm DQW active regions [26].

Alternatively, there is a known effective way for substantial improvement of hole injection uniformity in an MQW active region: the use of narrow barriers between the QWs. Such an approach was effectively applied to reduce efficiency droop in blue InGaN-based LEDs [112]. Therefore, to achieve a uniform carrier injection in the active region of UVC LDs with an acceptable optical confinement factor, a prospective solution is to substitute the conventional SQWs and DQWs with undoped GaN/AlGaN or AlGaN/AlN SPSLs with a high effective Al content. This approach has been successfully used in DUV-LEDs with SPSL active regions ~25–40 nm wide [86]. Since the SPSL active regions can be realized with thicknesses up to hundreds of nanometers, this would also result in remarkably higher optical confinement factors.

Another consequence of the rather small thicknesses of the p-type EBL and cladding layers and the necessity to use p^+^-GaN on top of the LD structure to form low-resistive Ohmic contact is the high optical losses caused by the penetration of the waveguide mode into the p^+^-GaN layer [28,106]. A prospective solution is to substitute the p^+^-GaN with a p-type GaN/AlGaN SPSL. On the one hand, the width of the GaN layer in SPSL may be chosen as sufficiently small to shift the absorption edge to wavelengths shorter than the oscillation one, thus avoiding strong band-to-band absorption of the waveguide mode. On the other hand, the p-type Ohmic contact can be formed to the top GaN material, providing the desired low resistance [89].

### 4.4. Tunnel Junctions in the Laser Diode Structures 

As it was mentioned in Section 3.2, the problem of insufficiently-high p-doping of AlGaN and the relevant problem of p-type Ohmic contact formation can be solved by using a low-resistive TJ placed on top of p-layers of an LD structure followed by an n-type spreading/contact AlGaN layer, resulting in a low-resistive Ohmic contact [113]. In the operating LD or LED, such a TJ is reverse-biased. In the case of homoepitaxial TJ, i.e., that made of the same material but n^+^- and p^+^-doped on the opposite sides of the junction, it is practically impossible to obtain a high hole density in p-side of TJ (see Section 3.2). This leads to the formation of a wide spatial-charge region on this side, resulting in extremely low probability of band-to-band tunneling and, eventually, in a high specific resistance of TJ [114]. 

To overcome this problem, insertion of narrow-bandgap materials such as InGaN between two wide-bandgap n^+^- and p^+^-AlGaN cladding layers was suggested to improve the TJ resistance [115]. The inserted layer, typically 4 nm In_0.25_Ga_0.75_N, worked as a polarization dipole, shifting the conduction and valence bands of the claddings towards each other to provide their better overlap. As a result, the band-to-band tunneling probability increases substantially, lowering the TJ resistance [115]. This approach was demonstrated to work effectively in AlGaN with low content of Al. However, the increase of the Al content up to 65–75% resulted in a remarkable increase of TJ resistance, producing extra operating voltage of ~3–6 V at the current density of 1 kA/cm^2^ [116]. The use of a GaN insertion between AlGaN claddings instead of InGaN was demonstrated in [117]. To date, the specific resistances of TJs suitable for DUV-LDs lie in the range of ~2–6 × 10^−3^ Ω·cm^2^, which is still insufficiently low for the operation currents of ~10 kA/cm^2^ and above. Further reduction of the TJ resistance is associated with using DPD [118] and selective doping of individual TJ layers.

Another specific problem for both p-doping and for the TJs is the passivation of Mg acceptors with hydrogen during the MOVPE-grown AlGaN [114]. It is believed that hydrogen incorporation efficiency increases remarkably with the Al content in the alloys, which may result in a partial acceptor activation by thermal annealing after growth. This aspect, however, has not been yet investigated in great detail. In contrast, the MBE is free of this problem since H_2_ carrier gas is not used during the growth with this technique.

## 5. Summary 

In this review, we have discussed the main issues faced in the development of efficient UVC LDs. Most of the problems originate from the intrinsic properties of AlGaN alloys and AlGaN-based heterostructures. In particular, the AlGaN structures are found to be less tolerant to generated TDD than InGaN/GaN LED and LD structures. Effective approaches aimed at reducing TDD in Al-rich AlGaN heterostructures are reviewed in this paper. Among them, high-temperature face-to-face annealing appears to be the most attractive solution due to the low TDD, and it has been already demonstrated, with relatively simple implementation. Since TDD is the property that largely controls the threshold current density of a UVC LD, and growth of device structures is carried out on a thick AlN buffer layer, a compromise should be found between the desirable TDD reduction and the possibility of stress relaxation in the LD structure during growth, also controlled by TDD. Finding of such a compromise is one of the tasks for future studies.

Limitations of n- and p-type doping in Al-rich AlGaN alloys are considered in view of obtaining heavily-doped cladding layers in LD structures. The electron concentration is now limited by self-compensation of Si donors tentatively attributed to the massive generation of point defects. To date, the maximum electron concentration attained in Al-rich AlGaN alloys is ~3–4 × 10^19^ cm^−3^. In contrast, the maximum hole concentration reached in p-type materials is ~1–2 × 10^18^ cm^−3^, with some exceptions of still-unclear origin. That is why the most effective approach, which provides a hole concentration sufficient for device operation, is based on DPD in graded-composition AlGaN alloys. Currently, the p-type DPD layers are routinely used for both hole generation in the claddings and partial suppression of electron leakage into p-sides of LD structures. The design of DPD layers still requires optimization aimed at avoiding their cracking during growth of the epitaxial structure.

An alternative solution for the p-doping problem is the use of TJs on top of p-layers followed by the deposition of the n-type AlGaN spreading/contact layer. However, the attempts to use this approach in deep-UV light emitters have resulted in very high specific resistance of the TJs, negatively affecting the device performance. A solution for this problem is critically needed to make the use of TJs in deep-UV lasers practical.

Practically unavoidable in the metal-polar (0001) LD structures is electron leakage into the p-type layers, which is attributed to the small conduction-band offsets between active regions and EBLs. The approaches to leakage suppression developed to date are not very effective to resolve the problem completely. On the other hand, the use of N-polar LD structures seems to be promising in view of natural high potential barriers formed by EBLs due to the inversion of polarization charge signs at the interfaces of such structures. The implementation of the latter approach would require improvement of current growth technologies capable of fabricating N-polar structures.

Using SPSL in the DUV-LDs may also improve their performance. First, SPSL design of the active region seems to be capable of improving uniformity of hole injection and increasing the active region width without losses of optical gain. As a result, the optical confinement factor may be risen substantially, thus lowering the threshold and operating currents of the lasers. Second, p^+^-type GaN/AlGaN(AlN) SPSL with a top GaN layer may serve as a low-resistive low-absorbing contact layer alternative to the currently-used p^+^-GaN.

To date, the first stage of development of electrically-driven UVC LDs has been completed with demonstration of room-temperature continuous-wave operation of a laser emitting at 274 nm [26]. The next stage seems to be focused on considerably improving the LD performance and, first of all, on further lowering of the threshold/operating current density and increasing of the slope efficiency. This task looks very challenging, as the room for further performance improvement of the conventional (0001) LD structures is largely limited by the intrinsic properties of Al-rich AlGaN alloys. Therefore, changes in the existing development paradigm are expected. In particular, the use of N-polar or a non-/semi-polar crystal orientation may be helpful to overcome the problem of electron leakage into p-sides of the LD structures. This and other novel approaches still require their practical validation and implementation. 

## Data Availability

Not applicable.
